# Metformin Induces Apoptosis and Inhibits Notch1 in Malignant Pleural Mesothelioma Cells

**DOI:** 10.3389/fcell.2020.534499

**Published:** 2021-01-18

**Authors:** Marika Rossini, Fernanda Martini, Elena Torreggiani, Francesca Fortini, Giorgio Aquila, Francesco Vieceli Dalla Sega, Simone Patergnani, Paolo Pinton, Pio Maniscalco, Giorgio Cavallesco, Paola Rizzo, Mauro Tognon

**Affiliations:** ^1^Department of Medical Sciences, University of Ferrara, Ferrara, Italy; ^2^Laboratory for Technology of Advanced Therapies (LTTA), University of Ferrara, Ferrara, Italy; ^3^Surgery Unit, Sant’Anna University Hospital, Ferrara, Italy; ^4^Department of Morphology, Surgery and Experimental Medicine, Section of Pathology, Oncology and Experimental Biology, University of Ferrara, Ferrara, Italy

**Keywords:** malignant pleural mesothelioma, metformin, cell proliferation, apoptosis, NOTCH1

## Abstract

Malignant pleural mesothelioma (MPM) is an aggressive asbestos-related cancer arising from the mesothelial cells lining the pleural cavity. MPM is characterized by a silent clinical progression and a highly resistance to conventional chemo/radio-therapies. MPM patients die in a few months/years from diagnosis. Notch signaling is a well-conserved cell communication system, which regulates many biological processes. In humans, the dysregulation of Notch pathway potentially contributes to cancer onset/progression, including MPM. Metformin is the first-line drug used to treat type 2 diabetes mellitus. Metformin is proven to be an effective antitumor drug in preclinical models of different types of cancer. To date, clinical efficacy is being studied in many clinical trials. In this study, the anti-proliferative effect of metformin on MPM cells and the putative involvement of Notch1 as a mediator of metformin activities, were investigated. MPM cells showed high levels of Notch1 activation compared to normal pleural mesothelial cells. Furthermore, metformin treatment hampered MPM cell proliferation and enhanced the apoptotic process, accompanied by decreased Notch1 activation.

## Introduction

Malignant pleural mesothelioma (MPM) is an aggressive tumor, representing the most common primary malignancy affecting the pleura, which accounts for approximately 80% of mesothelioma cases. MPM has an estimated number of about 30,000 deaths each year worldwide (Furuya et al., 2018). Several epidemiological studies demonstrated that asbestos is the cause of MPM in workers with a history of occupational fibers exposure in which the disease develops after a long latency period, in the range of about 25–70 years, from asbestos exposure (Carbone et al., 2012). MPM is histologically heterogeneous including three main subtypes: (i) epithelioid, (ii) sarcomatoid, and (iii) the mixed or biphasic type (Husain et al., 2018). The presence of different MPM subtypes, with related aggressive biological behaviors and rates of response to treatment, is an important factor in the selection of a therapeutic strategy.

A large number of studies conducted in the last two decades has led to the identification of several biological processes that are dysregulated and may play a significant role in its development (Rossini et al., 2018). These alterations include dysregulated activity of growth factors and their specific receptors (Chia et al., 2019), apoptosis inhibition (Amoroso et al., 2016), alteration of cellular (Balatti et al., 2011) and circulating microRNAs (Bononi et al., 2016) and remodeling of intracellular calcium homeostasis and Ca^2+^-dependent pathways (Patergnani et al., 2015).

*In vitro* and *in vivo* studies, have also shown an association between MPM and the oncogenic simian virus 40 (SV40), suggesting a transforming synergistic action between asbestos fibers and SV40 (Bocchetta et al., 2000; Carbone et al., 2008; Mazzoni et al., 2012; Rotondo et al., 2019). Furthermore, mutations in specific genes have been associated to the development of MPM, such as germline mutations/inactivations discovered in the tumor suppressor gene BRCA1-associated protein 1 (BAP1) in cases with a family history of cancer (Testa et al., 2011; Carbone et al., 2019). At present, there is no effective cure for MPM. There is therefore a growing interest in identifying novel approaches for early detection and an effective therapy for this deadly cancer. Metformin is the current first-line drug used in the treatment of type 2 diabetes mellitus (T2DM), with more than 120 million treated patients worldwide (Zi et al., 2018). Patients with untreated diabetes and T2DM have an increased cancer risk, attributed mostly to the growth-promoting effect of chronic elevated plasma glucose and insulin levels (Giovannucci et al., 2010; Noto et al., 2012). Insulin resistance and resultant hyperinsulinemia might indeed promote carcinogenesis directly through the insulin receptor or indirectly by increasing the levels of insulin-like growth factor (IGF). The interest in potential anti-neoplastic and cancer preventive properties of metformin is based on numerous clinical studies that showed a significantly reduced incidence of neoplastic diseases and cancer mortality in diabetic patients treated with metformin compared to diabetic patients treated with other antidiabetic drugs (Mansouri and Mahmoodi, 2017). A recent study performed in T2DM patients found no association between treatment with metformin and survival in MPM patient. However, this retrospective cohort study was conducted with some restrictions and limitations represented by a small sample size of pleural or unspecified mesothelioma cases, and by missing data for tumor stage, histological subtype and smoking status. This means that the association between metformin treatment and survival to mesothelioma may be underestimated (Wu et al., 2016).

There is evidence of a metformin-mediated regulation of Notch (Chen et al., 2015), a pathway dysregulated in MPM, thus suggesting a role for Notch in this cancer (Bocchetta et al., 2003). The Notch signaling pathway, a highly conserved evolutionary system involved in short-range intracellular communication, plays many key roles in the regulation of cell proliferation and survival (Bigas and Espinosa, 2018). In canonical Notch signaling, Notch ligands (i.e., Delta-like 1,4 and Jagged 1,2) bind to their receptors (Notch 1-4) in neighboring cells (Siebel and Lendahl, 2017) triggering an enzymatic cut, releasing Notch Intracellular Domain (NICD), which transfers to the nucleus to regulate target genes (Bray and Gomez-Lamarca, 2018; Zi et al., 2018). Dysregulation of Notch in cancer onset/progression has been extensively investigated (Rizzo et al., 2008b; Kushwah et al., 2014; Brzozowa-Zasada et al., 2017). Specifically, elevated Notch-1 and reduced Notch-2 expression have been observed in mesothelioma cells compared to normal mesothelial cells (HM) (Graziani et al., 2008). Notch inhibition as potential approach to stop cancer progression is being investigated in several types of tumors (Takebe et al., 2015; Tamagnone et al., 2018) and could represent also a new therapeutic strategy for MPM.

## Materials and Methods

### Cell Cultures

Human MPM cell lines, MMP89 (sarcomatoid histotype) and IST-Mes2 (epithelioid histotype), obtained from the GMP Cells and Cultures Bank, National Cancer Institute (ICLC, Genoa, Italy), were grown in DMEM Ham’s F12 (Lonza, Basel, Switzerland) supplemented with 10% fetal bovine serum, FBS (EuroClone, Milan, Italy). Primary pleural mesothelial cells (HM) were obtained from biopsies collected from non-oncologic patients affected by pneumothorax at the Surgical Clinic of the University/Hospital of Ferrara, Department of Thoracic Surgery. The study was approved by the County Ethics Committee, Ferrara. All subjects gave written informed consent in accordance with the Declaration of Helsinki. HM cells were grown in RPMI-1640 medium, 2 mM L-Glutamine (Lonza), supplemented with 10% FBS (EuroClone). All cell lines were maintained in their respective media supplemented with antibiotics (10,000 U ml^–1^ penicillin, 10,000^–1^ streptomycin) (Lonza), incubated at 37°C in 5% CO_2_-humidified atmosphere. Each experiment was performed at 80–90% confluence. HM cells and MPM cell lines were characterized at mRNA for the expression of the following markers: calretinin, CALB2; mesothelin, MSLN; keratin 7, KRT7 and high mobility group protein B1, HMGB1. The molecular analysis was performed using quantitative Real-Time PCR assay (Supplementary Figure 4).

### Chemicals

Metformin and N-[N-(3,5-difluorophenacetyl-l-alanyl)]-S-phenylglycine t-butyl ester (DAPT) were purchased from Sigma-Aldrich (Milan, Italy). Metformin was resuspended in phosphate buffered saline, PBS (Lonza) to make a 1 M stock solution. DAPT was dissolved in dimethyl sulfoxide (DMSO) (Sigma-Aldrich) to make a 5 mM stock solution. DAPT was then freshly diluted to the desired concentration with culture medium. The final concentration of DMSO was at 0.05% (v/v).

### Cell Viability Assay

Malignant pleural mesothelioma and HM cells (6 × 10^3^ well^–1^) were seeded in 96-well plate and treated with different concentrations of metformin, DAPT or their combination. For metformin experiments, MPM and HM cells were initially treated with 1, 5, and 10 mM of metformin (data not shown) and then with higher concentration, 25 and 50 mM, for 24 and 48 h. For DAPT experiments, MPM cells were treated with scalar doses of DAPT, ranging from 1 μM up to 50 μM, for 48 h. In addition, MPM cells were treated with a combination of metformin (25 mM) and a single concentration of DAPT (10 μM), for 24, 48, and 72 h. The Alamar Blue assay was used to quantitatively measure the effect of the drugs on cell viability. At 24, 48, and 72 h after drug treatment, cells were exposed to 5% of Alamar Blue solution (Invitrogen, Monza, Italy), and after 3 h of incubation, the absorbance was read at wavelengths of 570 nm and 620 nm using a microplate-reader (Sunrise Tecan, Männedorf, Switzerland). Data were reported as percentage of relative cell viability, based on the ratio of the absorbance of treated cells vs. control cells (set = 100%).

### Apoptosis Assay

Cell apoptosis was evaluated by Annexin V/Propidium Iodide (PI) assay. MPM and HM cells (1.0 × 10^6^ well^–1^) were seeded in six-well plates and treated with 25 mM metformin for 24 h. Briefly, cells were treated with metformin for 24 h, stained with Annexin V-FITC and PI (according to the manufacturer’s instruction, 100 ng/mL of Annexin V-FITC and 10 μg/mL of PI (Thermo Fisher Scientific, Waltham, MA, United States) in a binding buffer (10 mM HEPES, 5 mM KCl, 150 mM NaCl, 1.8 mM CaCl_2_, 1 mM MgCl_2_, pH 7.4), and then analyzed by flow cytometry (BD FACSCalibur, Becton-Dickinson Biosciences, San Jose, CA, United States), to quantify apoptotic and necrotic cells. Data analysis was performed with Kaluza Flow Analysis Software (Beckman Coulter, Milan, Italy). We considered Annexin V-positive/PI-negative as early apoptotic cells; Annexin V-positive/PI-positive as late apoptotic cells and Annexin V-negative/PI-positive as necrotic cells. The apoptosis rate was expressed as percentage of Annexin V-positive cells on total cells. Necrosis rate was expressed as percentage of PI-positive/Annexin V-negative cells on total cells.

### Immunofluorescence

Assessment of nuclear morphology by fluorescence microscopy was performed using as cell-permeable nucleic acid stain, the 4′,6′-diamidino-2-phenylindole (DAPI). Briefly, MPM cells (6 × 10^3^ well^–1^) were seeded on cover glasses and treated with 25 mM metformin. After 24 h, cells were fixed with 10% of neutral buffered formalin (Bio-Optica, Milan, Italy) for 7 min at room temperature. Following PBS washes, DAPI solution (Thermo Fisher Scientific) was added to cover glasses and incubated for 10 min at room temperature. After three PBS washes, two drops of mounting media were added to the cells, inverted and placed onto a glass slide. Images were acquired with an Eclipse E-2000 fluorescence microscope (Nikon Instruments, Sesto Fiorentino, Italy) equipped with a DXM 1200 F digital camera, using the ACT-1 software (Nikon Instruments).

### Western Blot Analysis

Following treatment with metformin 25 mM for 24 and 48 h, MPM cells were collected and lysed in RIPA buffer (0.1% SDS, 1% NP40, sodium deoxycholate), supplemented with protease inhibitors (10 μg/ml aprotinin, 10 μg/ml leupeptin, 10 μg/ml pepstatin A, 1 mM phenylmethylsulfonyl fluoride (PMSF) and 1 mM sodium orthovanadate), purchased from Sigma-Aldrich. The concentration of protein lysates was quantified using the BCA protein assay Kit (Thermo Fisher Scientific). Proteins were separated on 4–12% SDS-PAGE (Invitrogen) and transferred onto PVDF membranes (GE Healthcare, Milan, Italy). For immunoblotting analysis, the PVDF membranes were blocked with 5% non-fat milk and incubated overnight at 4°C with following primary antibodies: anti-cleaved Notch1 (Val1744) (1:1000), anti-pRPS6 (Ser240/244) (1:1000), anti-Noxa (1:1000), anti-Bcl2 (1:1000), anti-caspase-3 (1:1000), anti-caspase-8 (1:1000), anti-caspase-9 (1:1000), anti-PARP (1:1000) (all purchased from Cell Signaling Technology, Danvers, MA, United States), anti-Notch1 (C-20) (1:500) (Santa Cruz Biotechnology, Inc., Dallas, TX, United States) and anti-β actin (1:10000) (Sigma-Aldrich). After washing with Tris buffered saline (TBS)-Tween 20, the membranes were incubated with goat anti-mouse (1:3000) or goat anti-rabbit (1:3000) secondary antibodies both conjugated to horseradish peroxidase (Invitrogen). Immunocomplexes were detected using the Pierce ECL western blotting substrate (Thermo Scientific, Waltham, MA, United States) and visualized by Image Lab Software 4.0 (Bio-Rad, Milan, Italy). Band intensity was also quantified by Image Lab Software 4.0 (Bio-Rad).

### Statistical Analysis

Statistical analysis was performed by GraphPad Prism 6 Software (GraphPad Software, La Jolla, CA, United States) using both one-way analysis of variance and two-way ANOVA. The unpaired *t* test was used to compare differences between selected groups. Data were expressed as the mean ± SEM. *P* < 0.05 was considered as statistically significant.

## Results

### Metformin Inhibits MPM Cell Viability in a Dose- and Time-Dependent Manner

**FIGURE 1:**
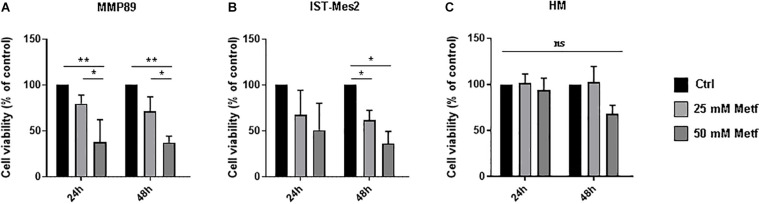
Metformin inhibited the MPM cell viability. **(A)** Cell viability of MMP89, **(B)** IST-Mes2, and **(C)** human normal mesothelial (HM) cells after treatment with 25 or 50 mM metformin for 24 and 48 h. Specifically, the significativity of 50 mM Metformin vs. Control and 50 mM Metformin vs. 25 mM Metformin, in HM cells at 48 h, were *P* = 0.0512 and *P* = 0.0538, respectively. Cell viability was determined by Alamar Blue assay. The results are shown as percentage relative to untreated control for each treatment group. Data represent mean ± SEM of three independent experiments in duplicate (*P < 0.05 and **P < 0.01, compared to untreated control). Metf: Metformin; ns: no statistically significant.

To investigate the effects of metformin on MPM cells, we assessed cell viability using the Alamar Blue assay. In the first step of our experiment, a range of metformin concentrations (1, 5, 10, 25, and 50 mM) was tested to defined the minimum metformin dose that affects the viability of two different MPM cell lines (MMP89 and IST-Mes2) following 24, 48, or 72 h (data not shown). Concentrations of 25 and 50 mM metformin, were identified as sufficient doses to efficiently decrease MPM cell viability without affecting normal human mesothelial (HM) cell viability. Specifically, after treatment with 25 mM metformin for 24 h and 48 h, MMP89 cell viability was reduced to 79.7% (±9.3) and 71.1% (±15.9), respectively. Treatment with 50 mM metformin for 24 and 48 h, reduced cell viability to 37.4% (±12.3) and 37.3% (±6.9), respectively (Figure 1A). IST-Mes2 cells treated with 25 mM metformin for 24 and 48 h showed a decrease of the cell viability to 67.6% (±26.6) and 61.9% (±10.5), respectively, whereas treatment with 50 mM metformin resulted in to 50.3% (±29.1) and 36.1% (±13.3) reduction, respectively (Figure 1B). Under the same experimental conditions, metformin was not cytotoxic for normal human mesothelial (HM) cells (Figure 1C). Overall, these results showed that treatment with 25 mM metformin decreased MPM cells viability without affecting growth of HM cells, hence, this concentration was chosen for the following experiments.

### Metformin Induces Apoptosis in MPM Cells

**FIGURE 2:**
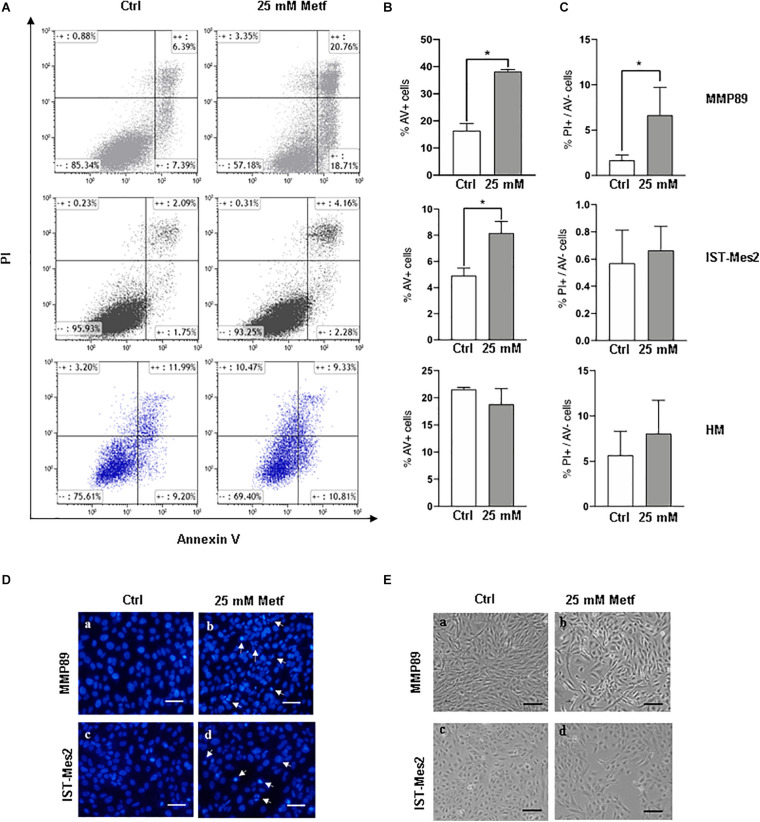
Metformin induces apoptosis in MPM cells but not in HM cells. **(A)** Representative flow cytometry plots for apoptosis assessment in MMP89, IST-Mes2, and HM following 24 h of 25 mM metformin treatment. **(B)** Graph bars show a significant increase of Annexin V-positive MMP89 and IST-Mes2 cells after metformin treatment, while HM cells did not show any significant increase. Results are the mean ± SEM of three experiments (*P < 0.05 compared to untreated cells). **(C)** Graph bars show that percentage of MMP89 necrotic cells (PI+/Annexin V–), but not IST-Mes2 and HM necrotic cells, increases after 24 h of 25 mM metformin treatment. Results are the mean ± SEM of three experiments. **(D)** Nuclear staining with DAPI solution of (a,c) untreated MPM cells and (b,d) metformin-treated cells at 24 h. Images were captured under fluorescence microscope. Arrows indicate cells with apoptotic nuclei. **(E)** Representative images shows morphological changes of MPM cells following 24 h of (b,d) metformin treatment compared with (a,c) untreated cells. Images were captured under inverted phase contrast microscope (10× magnification). Scale bar: 25 μm. AV: Annexin V; PI: propidium iodide; Metf: Metformin.

The effects of metformin treatment, were then evaluated on MPM cell apoptosis by flow cytometric analysis following Annexin V/PI staining. Following incubation with 25 mM metformin for 24 h, MMP89 and IST-Mes2 Annexin V-positive cells were increased compared to the untreated cells. Importantly, metformin did not cause apoptosis of HM cells in the same experimental conditions (Figures 2A,B). Furthermore, MMP89 showed an increase of necrotic cells after metformin treatment. On the contrary, IST-Mes2 and HM cells did not show statistically significant differences on the necrotic cells number (Figure 2C).

The induction of apoptosis in metformin-treated cells was also assayed by analyzing morphological changes in cell nuclei by DAPI staining (Figure 2D). After metformin treatment, MPM cells appeared brighter, granular, blue fluorescent and showed typical changes of late apoptosis, such as small apoptotic bodies, compared to untreated cells (Figure 2D, panels b, d). Moreover, the apoptotic effect of metformin was confirmed through alterations in MMP89 and IST-Mes2 morphology following metformin exposure (Figure 2E and Supplementary Figure 5). In particular, treatment of MMP89 and IST-Mes2 with metformin for 24 h revealed typical apoptotic features, such as rounding, shrinkage and losing contact with adjacent cells (Figure 2E, panels b, d).

### Metformin Increases the Levels of Pro-apoptotic Proteins in MPM Cells

**FIGURE 3:**
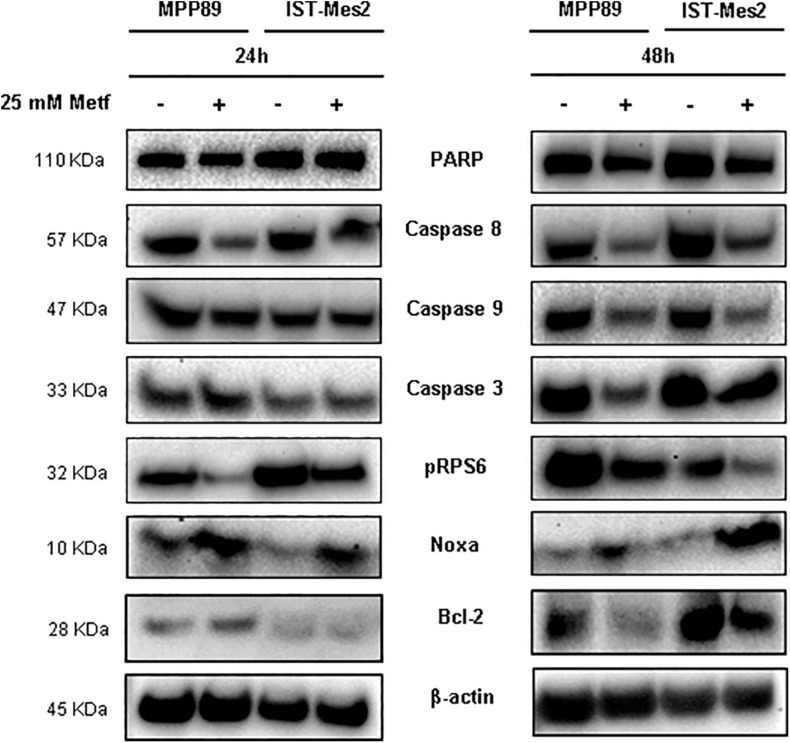
Induction of pro-apoptotic proteins after metformin treatment in MPM cells. Western blot analysis of PARP, Caspase 8, 9, and 3, pRPS6, Noxa and Bcl-2 in MPM cells after 24 and 48 h of 25 mM metformin treatment. β-actin was used as loading control. Protein molecular weights are reported (KDa).

We sought to investigate the molecular mechanisms underlying metformin-induced apoptosis in MPM cells by measuring the levels of caspases (3, 8, and 9), Bcl-2, PARP and Noxa proteins. The treatment with 25 mM metformin for 48 h caused an evident decrease of caspase expression, along with a down-regulation of PARP full length (inactive), up-regulation of Noxa and Bcl-2 down-regulation, in both MPM cell lines (Figure 3, right panel and Supplementary Figure 1). Conversely, the same treatment for 24 h induced only a slight decrease of caspase 8 and an increase of Noxa expression (Figure 3, left panel and Supplementary Figure 1). The anti-proliferative effect of metformin in some tumor cell types is due to mTOR inhibition (Foretz et al., 2014). Thus, mTOR activation in MPM cell was assessed by measuring the phosphorylation status of the mTOR down-stream target, S6 ribosomal protein (pRPS6) to confirm metformin efficacy in this context. Metformin at concentration of 25 mM caused a strong decrease in the phosphorylation of RPS6 protein in MPM cell lines after 24 or 48 h of treatment (Figure 3 and Supplementary Figure 1).

### Metformin Inhibits Notch1 and Its Anti-viability Effect Is Enhanced by DAPT in MPM Cells

**FIGURE 4:**
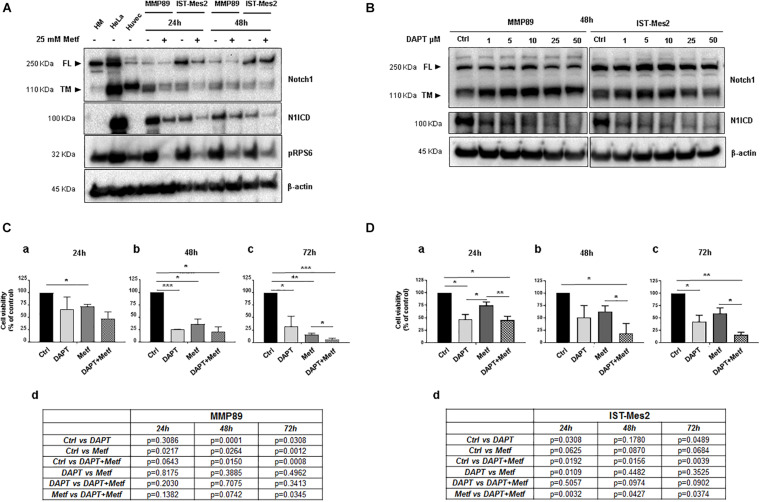
Effect of metformin and DAPT treatment on Notch1 activation and MPM cell viability. **(A)** Western blot analysis for Notch1 (full length, FL and transmembrane, TM) and its active form, cleaved Notch1, N1ICD. The blot shows up-regulation of N1ICD in untreated MPM cell lines compared with primary HM cells, in which Notch1 FL is expressed but not activated. Treatment with 25 mM metformin for 24 and 48 h down-regulated protein expression levels of Notch1 and its active form N1ICD, in both MPM cell lines. The human cervical cancer cells (HeLa) and human umbilical vein endothelial cells (Huvec) were used as positive controls for Notch1 (TM) protein and its active form. **(B)** Inhibition of Notch1 activation following DAPT treatment in MPM cells. MPM cell lines were treated with increasing concentrations of DAPT (1, 5, 10, 25, and 50 μM) for 48 h. Blot shows the inhibition of Notch1 (N1ICD) in MMP89 and IST-Mes2 for each concentration of γ-secretase inhibitor. β-actin was used as loading control. Protein molecular weights are reported (KDa). **(C,D)** Effect of DAPT-metformin combination on MPM cell viability. **(C)** MMP89 and **(D)** IST-Mes2 cells were treated with metformin 25 mM and/or 10 μM of DAPT for (a) 24, (b) 48, and (c) 72 h. 0.05% DMSO and PBS were added in the culture medium of the different experimental groups as vehicles. (d) Tables reported the *P*-value of all comparisons made. Error bars represent the mean ± SEM for three independent experiments (*P < 0.05, **P < 0.01, and ***P < 0.001 compared to untreated control). Metf: Metformin.

Noxa is inhibited by Notch1 (Rizzo et al., 2008b), which is activated and provides a pro-survival stimulus in mesothelioma (Graziani et al., 2008), hence we investigated whether inhibition of Notch1 could be involved in the pro-apoptotic activity of metformin. The Notch1 protein basal level was evaluated in both MPM cells. Full length (FL) and transmembrane (TM) Notch1 protein were detected by a specific antibody directed against the C-terminal of Notch1. The active form of Notch1 (N1ICD) was detected with an antibody specific for Valine 1744 at a cleavage site. Both MPM cell lines showed increased levels of N1ICD compared to normal human mesothelial cells, HM (Figure 4A and Supplementary Figure 2). Then, MMP89 and IST-Mes2 cells were treated with 25 mM metformin, for 24 and 48 h, followed by western blot analysis to assess the levels of N1ICD and of the transmembrane form (TM) of Notch1. Metformin treatment down-regulated N1ICD and Notch1 TM protein levels compared to untreated controls in both MPM cells (Figure 4A and Supplementary Figure 2). These data suggest that Notch1 inhibition was associated to a time-dependent, anti-viability effect of metformin.

Based on the results showing anti-proliferative effects and inhibition of Notch1 by metformin in MPM cells, we investigated whether Notch1 inhibition by DAPT would sensitize MPM cells to metformin-reduced cell viability. First, MMP89 or IST-Mes2 cells were treated with increasing DAPT concentrations, and then N1ICD levels were evaluated by western blots to determine the lowest DAPT concentration able to inhibit Notch1 activation. Treatment with 10 μM DAPT for 48 h strongly decreased N1ICD in both cell lines, hence this DAPT concentration was used for following experiments (Figure 4B and Supplementary Figure 3). MMP89 and IST-Mes2 cells were pre-treated with 10 μM DAPT for 24 h and then treated with metformin 25 mM for 24, 48, and 72 h. Cell viability was assessed by Alamar Blue assay for both MPM cell lines (Figures 4C,D). When MPM cells were co-treated with DAPT and metformin, there was a time-dependent decrease of cell viability, compared to metformin alone. Specifically, the concentration of 25 mM metformin inhibited MMP89 cell viability to 72.2% (±2.4), 36% (±10.6), and 16.2% (±2.8), respectively, after 24, 48, and 72 h of treatment (Figure 4C). In the same conditions, IST-Mes2 cells viability was inhibited to 74.7% (±6.6), 62.2% (±11.9), and 59.2% (±11.2), respectively (Figure 4D). When MPM cells were treated with 25 mM metformin and 10 μM DAPT, an enhancement of the metformin effect on cell viability decrease was observed in both MPM cell lines and in a time-dependent manner. In particular, MMP89 cells exposed to DAPT and treated with metformin for 72 h showed a significant decrease in cell viability compared to cells treated with metformin alone (Figure 4C). Conversely, IST-Mes2 incubated with DAPT and metformin exhibited a significant reduction of cell viability compared to metformin treated cells, at each time point tested (Figure 4D).

## Discussion

Malignant pleural mesothelioma prevalence increased in recent years (Robinson, 2012), representing an enormous burden on the health system worldwide, particularly in light of the presence of environmental asbestos fibers (Baumann et al., 2015; Noonan, 2017). Thus, there is urgent need for new therapeutic approaches against this deadly disease. Epidemiological evidence show that diabetes is strongly associated with cancer (Noto et al., 2012; Mansouri and Mahmoodi, 2017) and retrospective analyses have shown that metformin, a drug used to treat T2DM, possesses antineoplastic properties since it increases survival in diabetic patients affected by several types of tumors with less toxicity compared to existing anti-cancer drugs (Zi et al., 2018). To date, little is known on the effect of metformin treatment on MPM progression. In our study, the effects of metformin were tested in MPM cell lines. We found that metformin reduced viability and increases apoptotic rate of MPM cells in a dose- and time-dependent manner, with MMP89 cells being more sensitive to the apoptotic action of this drug compared to IST-Mes2 cells (Figures 1, 2). The molecular mechanisms underlying the different response of the two cell lines to metformin remain to be investigated. Our results are in agreement with previous investigations reporting similar effects of metformin in other tumor cell lines (Rattan et al., 2011; Ma et al., 2019) and show, for the first time, that metformin arrests cell growth and induces apoptosis in MPM cells. In MPM cells treated with metformin we found inhibition of mTOR and induction of a pro-apoptotic profile, with activation of caspases, inhibition of Bcl-2 after 48 h of treatment, whereas an increase of Noxa expression was already observed after 24 h (Figure 3 and Supplementary Figure 1). These results suggest that metformin induces an up-regulation of Noxa, which *per se* entailed significant cell death (Figure 2), as demonstrated in some systems (Oda et al., 2000) and this increase is accompanied by cytochrome c release and subsequent caspase activation (Ploner et al., 2008). In order to demonstrate that metformin induces a caspases-depend cell death, experiments with various peptide inhibitors, either directed against individual caspases, or the zVAD-fmk broad-spectrum inhibitor will be performed. The inhibition of caspase enzymatic activity by zVAD-fmk in human renal cell carcinoma A498 cells, demonstrated that metformin-induced apoptosis facilitated degradation of the cellular caspase 8 (FLICE)-like inhibitory protein and activated procaspase-8 through a caspase-dependent pathway (Jang et al., 2018). In addition, the employment of caspase inhibitors in breast cancer cells, revealed that metformin induces both caspase-dependent and poly(ADPribose) polymerase-dependent cell death (Zhuang and Miskimins, 2011). In addition to Noxa up-regulation, MPM cells treated with 25 mM metformin, showed an inhibition of Notch1 (Figure 4A and Supplementary Figure 2), a known suppressor of Noxa expression in cancer cells (Rizzo et al., 2008a; Xu et al., 2012). In both MPM cell lines, Notch1 was up-regulated, compared to normal human mesothelial cells, HM (Figure 4A), and Notch1 inhibition by DAPT, an inhibitor of γ-secretase enzymatic activity (Bray and Gomez-Lamarca, 2018), decreased their viability (Figures 4B–D). Activation of Notch1 in mesothelioma specimens has been previously reported by Graziani et al. (2008), which also showed that in mesothelioma cell lines Notch1 promotes cell proliferation and activates the pro-survival phosphatidylinositol 3-kinase (PI3K)/Akt/mammalian target of rapamycin (mTOR) signaling pathway by inhibiting PTEN. Song et al. (2015) reported that Notch1 inhibitor DAPT induced the adipogenic differentiation of bone marrow mesenchymal stem cells by activating early autophagy through the PTEN-PI3K/Akt/mTOR pathway. Recently, Hibdon et al. (2019) showed an interaction between Notch1 and mTOR signaling pathways in human gastric cancer. In particular, they demonstrated that cells treated with DAPT showed a decrease in the amount of pRPS6 phosphorylation, indicating that mTOR activity is Notch1 dependent and that mTOR activation is downstream of Notch1 signaling in gastric cancer cells. Thus, we may speculate that, in our experimental model, Notch1 inhibition may be part of the mechanism by which metformin and DAPT can inhibit mTOR signaling, leading to inhibition of growth and apoptosis of MPM cells. Thus, Notch1 inhibition may be part of the mechanism by which metformin inhibits mTOR and up-regulates Noxa, leading to inhibition of growth and apoptosis of MPM cells. Notch1 activation has been observed in mesothelioma and in many other solid tumors and leukemias (Rizzo et al., 2008b) and, a large number of studies *in vivo* has demonstrated that up-regulation of the Notch1 signaling in cancer cells, reduce their sensitivity to commonly used apoptosis-inducing drugs, thus promoting resistance to these agents (Rizzo et al., 2008b; Espinoza and Miele, 2013). Inhibition of Notch1 by γ-secretase inhibitor (GSIs), in combination with commonly used anti-cancer drugs, is being investigated in clinical trials for different types of cancer (Takebe et al., 2015; Tamagnone et al., 2018). Given the major role played by Notch1 in maintaining the stem cells pool in renewing tissues (Koch et al., 2013), targeting of Notch in cancer has been hampered by gastrointestinal toxicity. Furthermore, Notch is a major regulator of immune system (Minter and Osborne, 2012; Vijayaraghavan and Osborne, 2018) and the effects of GSIs on the immunity are still largely unexplored. Thus new approaches aimed to block Notch1 in cancer cells without affecting the immune system, are being investigated (Hossain et al., 2018). We report that metformin was able to inhibit Notch1 and to block MPM cells growth and promote apoptosis. Of note, treatment with γ-secretase inhibitor DAPT and metformin was more effective than treatment with each agent alone in arresting MPM cells growth. In particular, this effect was more evident in IST-Mes-2 cells compared to MMP89 cells (Figures 4C,D). The different response of the two MPM cell lines to the co-treatment might be due to the different sensitivity of these cells to DAPT and to the different expression of Notch1 protein baseline levels. As shown in Figure 4A and Supplementary Figure 2, MMP89 cells have Notch1 basal level significantly higher compared to IST-Mes-2 cells. Probably, the higher expression of Notch1 in MMP89 cells make them less sensitive to 10 μM DAPT pre-treatment for 24 compared to IST-Mes-2 (see panel a, Figures 4C,D, respectively). Consequently, the combination of DAPT and metformin slightly affect MPP89 cell viability, while this effect is more evident in IST-Mes-2 cells. Since DAPT can affect several cellular pathways other than γ-secretase (Ran et al., 2017), more studies are needed to investigate the molecular mechanism by which metformin inhibits Notch1 and which could be at the basis of the different response of MMP89 and IST-Mes-2 cell lines. In particular, it would be interesting to perform pharmacological interaction studies to establish whether metformin and GSI work synergistically or additive in interfering with MPM cells growth. It is thought that metformin variability observed in clinical trials for distinct cancer treatments is due to its different concentrations that are achievable in tissue (ClinicalTrial.gov). Additionally, as with GSIs, there is concern with the side effect profile of metformin: even though clinical trials have revealed a low incidence of dose limiting toxicity with metformin in combination with a wide variety of chemotherapy regimens, metformin is well known for causing gastrointestinal problems (Chae et al., 2016). Our study, by showing that Notch1 inhibitors could act downstream or in parallel to metformin in inhibiting viability proliferation and inducing apoptosis of MPM cells, provides new data on a novel therapeutic strategy based on the combination of these two agents to treat MPM. Specifically, our results could lead to the identification of a therapeutic approach based on a combination treatment of metformin and GSI, able to block cancer cells with minimum side effects.

Mesothelioma is characterized by the presence of infiltrating M2 macrophages characterized by immunosuppressive function and whose levels represent a negative prognostic factor (Burt et al., 2011). Studies *in vitro* have instead reported an enhancement of M2 macrophages following metformin treatment (Hans et al., 2019). Nevertheless, in breast cancer, metformin has been reported to reduce the levels of M2 macrophages in favor of M1 and this has been explained on the basis of cytokines production by cancer cells induced by metformin (Chiang et al., 2017). Studies *in vivo* will be required to test the effect of the combined treatment metformin/GSIs on TAM receptors in mesothelioma. This treatment could be especially relevant for heterozygous carriers of germline BAP1 mutation (Testa et al., 2011) more at risk of MPM development due to high levels of M2 in pleural fluid (Napolitano et al., 2016).

In conclusion, we report for the first time, that metformin inhibits Notch1 and arrests growth of MPM cells. The results presented in this study could lead to a novel therapy against MPM based on metformin, a drug currently used worldwide against the type 2 diabetes for over a century, and GSIs, a new class of molecule, which is giving promising results for cancer therapy by blocking Notch1 receptor.

## Data Availability Statement

The datasets generated for this study are available on request to the corresponding authors.

## Ethics Statement

The studies involving human participants were reviewed and approved by The County Ethics Committee, Ferrara. The patients/participants provided their written informed consent to participate in this study.

## Author Contributions

MR, MT, and PR: conceptualization. MR, FM, ET, FF, and GA: investigation and methodology. PM and GC: clinical records and specimens. MR, FM, ET, SP, FV, MT, and PR: formal analysis and data curation. MR, ET, MT, and PR: writing–original preparation. MT and PR: supervision. PP: discussion preparation and final revision. All authors contributed to manuscript revision, read, and approved the submitted version.

## Conflict of Interest

The authors declare that the research was conducted in the absence of any commercial or financial relationships that could be construed as a potential conflict of interest.
