# Supplementary material for: Metformin Induces Apoptosis and Inhibits Notch1 in Malignant Pleural Mesothelioma Cells
Source: Front Cell Dev Biol. 2021 Jan 18;8:534499. doi: 10.3389/fcell.2020.534499 (PMC7849608; doi:10.3389/fcell.2020.534499)
Supplement: Supplementary file 1 [file Data_Sheet_1.pdf]

Supplementary Figures

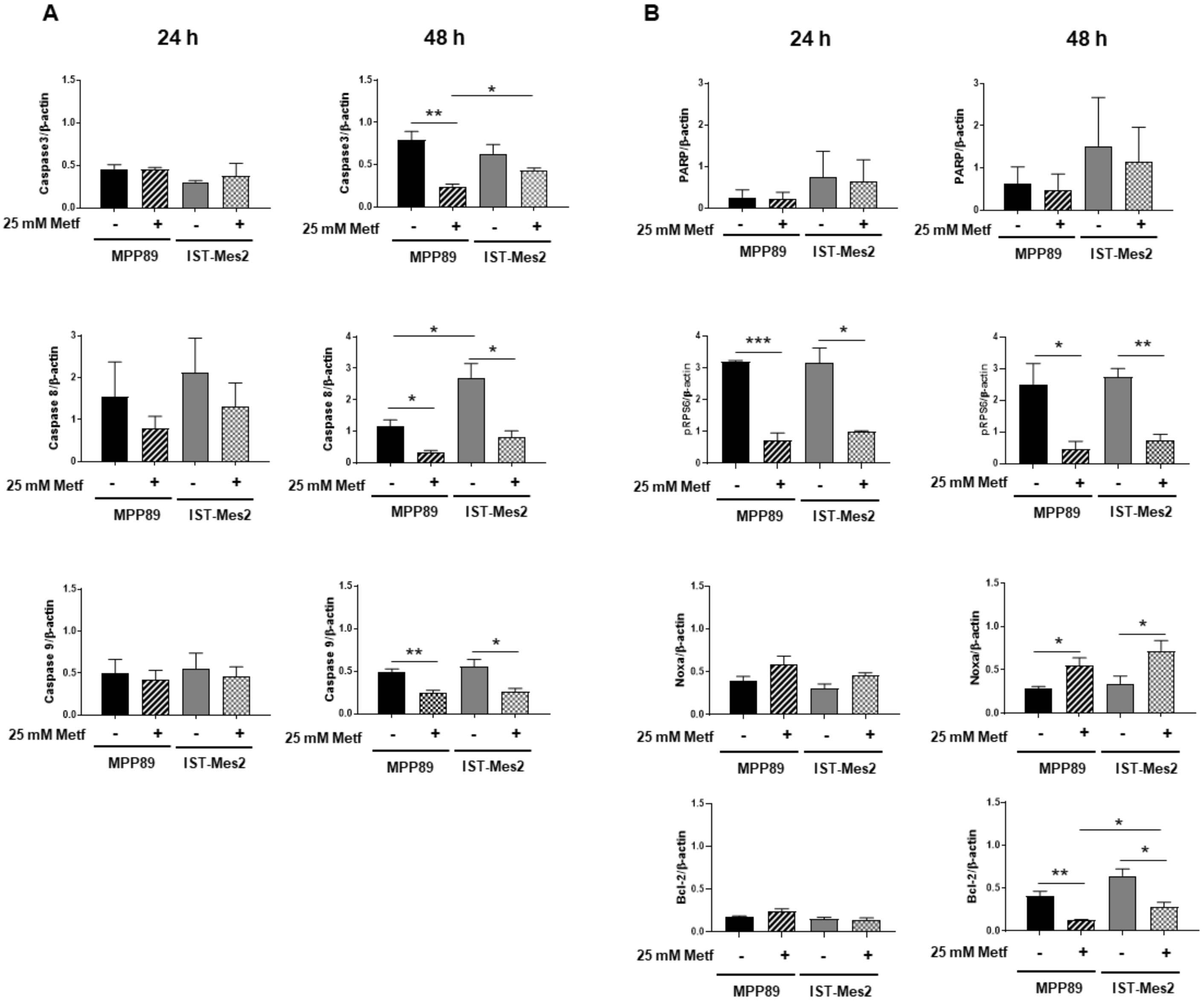

Supplementary Figure 1. Quantification of pro-apoptotic proteins after 24 h and 48 of 25 mM metformin treatment in MPM cells. Densitometric analysis of (A) Caspase 3, 8 and 9, (B) PARP, pRPS6, Noxa and Bcl-2 relative to β-actin (used as loading control). Band intensity was quantified by Image Lab Software 4.0. Values are expressed as means ± SEM for n = 3. *$p < 0.05$, **$p < 0.01$ and ***$p < 0.001$, compared to untreated control. Metf: Metformin.

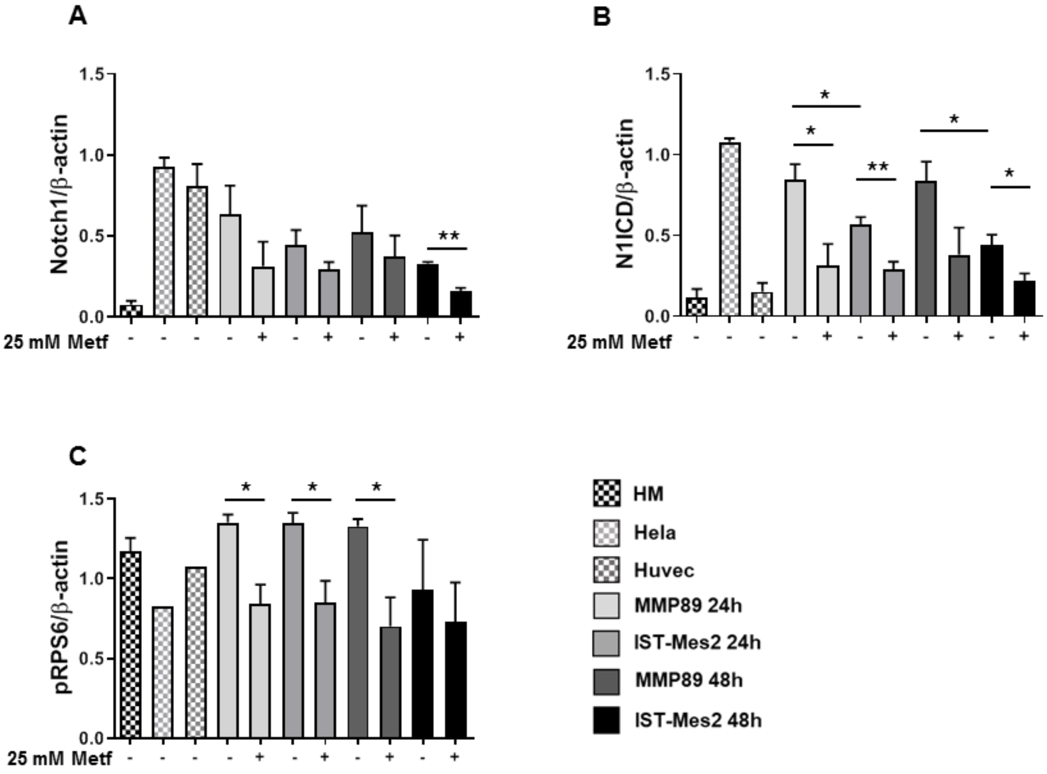

Supplementary Figure 2. Effect of metformin treatment on Notch-1 activation in MPM cells. Densitometric analysis of (A) the trans-membrane form of Notch1, (B) the active form of Notch1, N1ICD and (C) S6 ribosomal protein, pRPS6, relative to β-actin (used as loading control). Band intensity was quantified by Image Lab Software 4.0. Values are expressed as means ± SEM for n = 4. *p < 0.05 and **p < 0.01, compared to untreated control. Metf: Metformin.

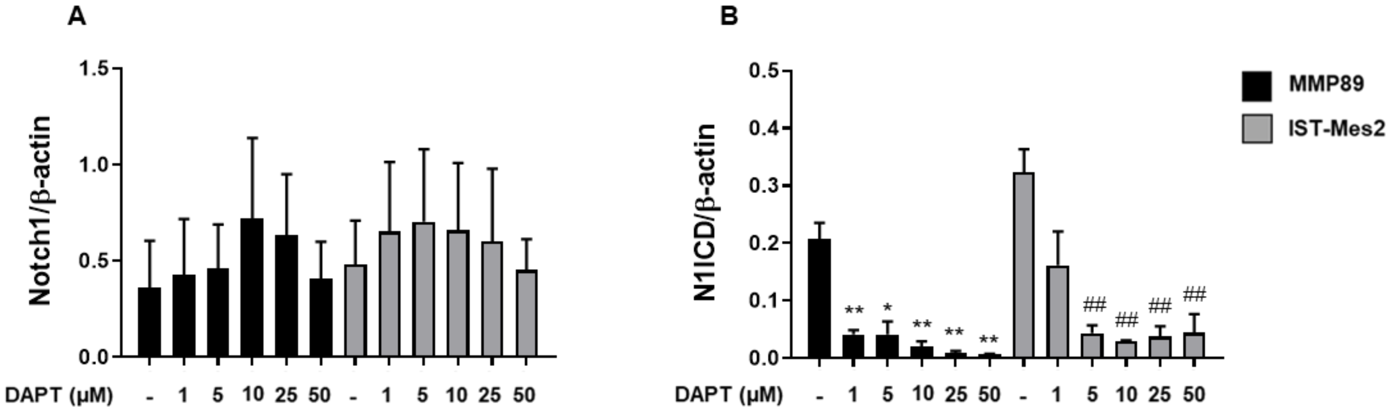

Supplementary Figure 3. Notch1 activation is inhibited by DAPT treatment. MPM cell lines were treated with increasing concentrations of DAPT (1, 5, 10, 25 and 50 μM) for 48 h. Densitometric analysis of (A) the trans-membrane form of Notch1 and (B) the active form of Notch1, N1ICD relative to β-actin (used as loading control). Band intensity was quantified by Image Lab Software 4.0. *p < 0.05, **p<0.001 versus Ctrl MMP89; ##p< 0.01 versus Ctrl IST-Mes2.Values are expressed as means ± SEM for n = 3. Metf: Metformin.

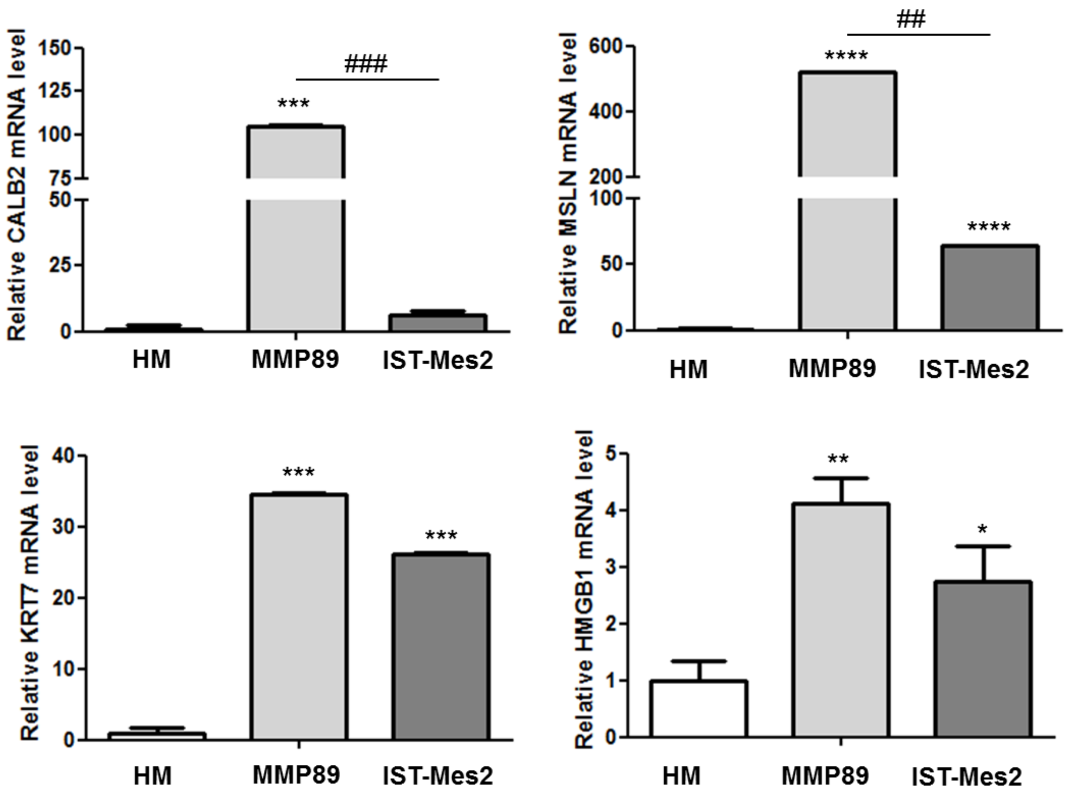

Supplementary Figure 4. HM and MPM cell lines characterization by qRT-PCR. CALB2, MSLN, KRT7 and HMGB1 expression were evaluated at mRNA level and revealed by quantitative RT-PCR analysis. qRT-PCR results were calculated using the ΔΔCt method, using GAPDH as the housekeeping gene. Statistical analysis was performed using one-way ANOVA and the unpaired t test. *$p < 0.05$, **$p<0.001$, ***$p < 0.001$, ****$p < 0.0001$ versus HM; ##$p< 0.01$, ###$p< 0.001$ MMP89 versus IST-Mes2. Values are expressed as means ± SEM for n = 3. CALB2: Calretinin; MSLN: Mesothelin; KRT7: Keratin 7; HMGB1: High Mobility Group Protein B1.

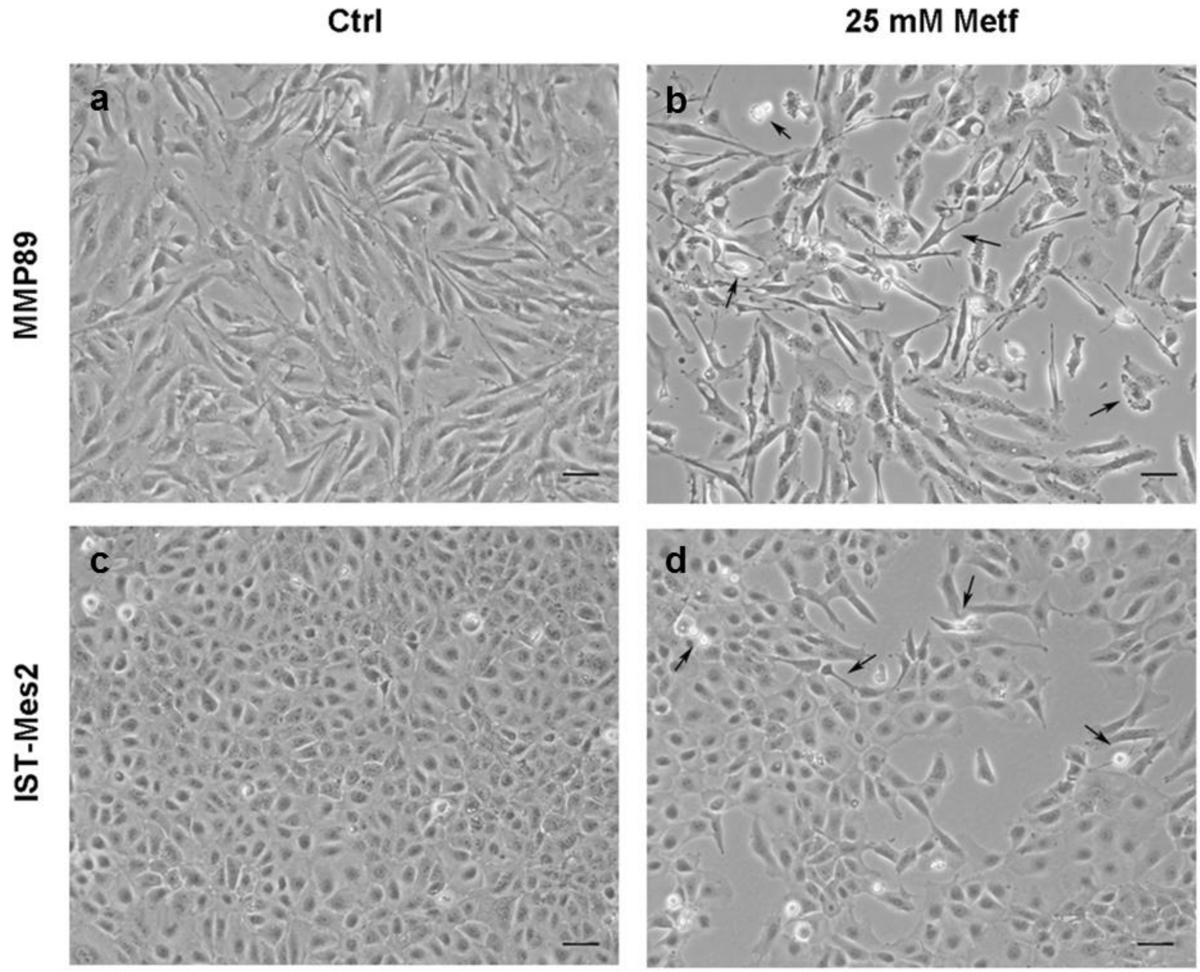

Supplementary Figure 5. Representative images shows morphological changes of MPM cells following 24 h of (b,d) metformin treatment compared with (a,c) untreated cells. Arrows indicates typical forms of morphological changes in apoptotic cells: cell shrinkage, membrane blebbing, apoptotic bodies, bubbling, and echinoid spikes. All images were captured under inverted phase contrast microscope (10x magnification). Scale bar: 25 μm. Metf: Metformin
